# aMMP-8 POCT vs. Other Potential Biomarkers in Chair-Side Diagnostics and Treatment Monitoring of Severe Periodontitis

**DOI:** 10.3390/ijms25179421

**Published:** 2024-08-30

**Authors:** Nur Rahman Ahmad Seno Aji, Ismo T. Räisänen, Nilminie Rathnayake, Fionnuala T. Lundy, Maelíosa T. C. Mc Crudden, Lata Goyal, Timo Sorsa, Shipra Gupta

**Affiliations:** 1 Department of Oral and Maxillofacial Diseases, Head and Neck Center, University of Helsinki and Helsinki University Hospital, 00290 Helsinki, Finland; nur.aji@helsinki.fi (N.R.A.S.A.); rathnayake.nilminie@helsinki.fi (N.R.); 2 Department of Periodontics, Faculty of Dentistry, Universitas Gadjah Mada, Jalan Denta No. 1, Sekip Utara, 10 Sleman, Yogyakarta 55281, Indonesia; 3 Wellcome-Wolfson Institute for Experimental Medicine, School of Medicine, Dentistry and Biomedical Science, Queen’s University Belfast, Belfast BT9 7BL, UK; f.lundy@qub.ac.uk (F.T.L.); m.mccrudden@qub.ac.uk (M.T.C.M.C.); 4 Periodontics Division, Department of Dentistry, All India Institute of Medical Sciences, Bathinda, Punjab 151001, India; latagoyal83@gmail.com; 5 Division of Oral Diseases, Department of Dental Medicine, Karolinska Institutet, 171 77 Stockholm, Sweden; 6 Oral Health Sciences Centre, Post Graduate Institute of Medical Education & Research, Chandigarh 160012, India; shipra1472@gmail.com

**Keywords:** periodontitis, aMMP-8, active matrix metalloproteinase-8, biomarker, diagnosis

## Abstract

This study aimed to compare several potential mouthrinse biomarkers for periodontitis including active matrix-metalloproteinase-8 (aMMP-8), total MMP-8, and other inflammatory biomarkers in diagnosing and monitoring the effects of nonsurgical periodontal therapy. Thirteen patients with stage III/IV periodontitis were recruited, along with thirteen periodontally and systemically healthy controls. These 13 patients were representative of the number of outpatients visiting any dentist in a single day. Full-mouth clinical periodontal parameters and biomarkers (the aMMP-8 point-of-care-test [POCT], total MMP-8, tissue inhibitor of MMPs (TIMP)-1, the aMMP-8 RFU activity assay, Myeloperoxidase, PMN elastase, calprotectin, and interleukin-6) were recorded at baseline and after nonsurgical therapy at 6 weeks. The aMMP-8 POCT was the most efficient and precise discriminator, with a cut-off of 20 ng/mL found to be optimal. Myeloperoxidase, MMP-8’s oxidative activator, was also efficient. Following closely in precision was the aMMP-8 RFU activity assay and PMN elastase. In contrast, the total MMP-8 assay and the other biomarkers were less efficient and precise in distinguishing patients with periodontitis from healthy controls. aMMP-8, MPO, and PMN elastase may form a proteolytic and pro-oxidative tissue destruction cascade in periodontitis, potentially representing a therapeutic target. The aMMP-8 chair-side test with a cut-off of 20 ng/mL was the most efficient and precise discriminator between periodontal health and disease. The aMMP-8 POC test can be effectively used by dental professionals in their dental practices in online and real-time diagnoses as well as in monitoring periodontal disease and educating and encouraging good oral practices among patients.

## 1. Introduction

Periodontitis is an infection-induced inflammatory condition that affects tooth-supporting tissues, causing irreversible connective tissue breakdown [1,2,3]. Periodontitis is classified in four stages (disease severity) and three grades (rate of progression) in the latest 2017 classification system of periodontitis: stage I (initial periodontitis), stage II (moderated periodontitis), stage III (severe periodontitis), and stage IV (advanced periodontitis with extensive tooth loss); grade A (slow), grade B (moderate), and grade C [2,3]. Treatment approaches of periodontitis are related to the disease stage and progression, and the interventions required increase incrementally as the disease stage advances, ranging from guiding behavioral changes, controlling risk factors, controlling gingival inflammation and supra- and subgingival biofilm to different types of periodontal surgical interventions [4,5].

The 2017 classification system of periodontitis and its framework allows the integration of validated biomarkers to increase the diagnostic accuracy of periodontitis [3]. Matrix metalloproteinase (MMP)-8, also known as neutrophil collagenase or collagenase-2, is among the key proteolytic enzymes involved in the destruction of tooth-supporting periodontitis-affected soft and hard tissues [6,7,8,9,10]. MMP-8 is also necessary for defensive immune responses [6,7,8,9,10] and tissue repair and remodeling, but its excessive degranulation by triggered neutrophils and concomitant activation in response to inflammation eventually lead to destruction and the progression of periodontal disease [7,10,11,12,13,14,15]. An emerging focus is now on use of active MMP-8 as a biomarker for diagnosing, grading, screening, and evaluating treatment responses in periodontitis [15,16,17,18,19,20]. Recently, an oral rinse point-of-care (POC) lateral flow immunoassay mainly detecting aMMP-8 has been developed, successfully substantiated and validated by clinical studies in several countries [18,21,22,23,24,25,26,27,28,29]. The active MMP-8 POC test (POCT) has been evidenced to be the most precise and superior relative to the other potential assayed biomarkers and clinical indices such as total MMP-8 and BOP [18,29,30]. This originally visual, chair-side test can also be used quantitatively by a computer reader online and in real-time to diagnostically stage and grade as well as screen for periodontitis [18,23,25,29,31]. The aMMP-8 biomarker was therefore deemed as being more precise and effective than the conventional BOP and total MMP-8 analysis, especially in its ability to identify subclinical periodontitis/pre-periodontitis [23,25].

Studies that involve measuring periodontal parameters from a few index teeth indeed can underscore the underlying inflammatory status, and the more comprehensive full-mouth periodontal parameter analysis involving all teeth is necessary, as some teeth with more periodontal breakdown can be unintentionally excluded [1,2,3]. In this regard, utilizing a simple, non-invasive biomarker test for mouthrinse aMMP-8 would be an invaluable adjunctive measure to periodontal pocketing of the index teeth and could possibly help to detect and predict more accurately the on-going and/or developing periodontal attachment tissue loss [18,23,29,32]. The observation of alarmingly elevated mouthrinse aMMP-8 levels in patients with periodontitis through both point-of-care aMMP-8 technology and time-resolved immunofluorometric assay (IFMA) aMMP-8 analyses, and their significant decrease after anti-infective periodontal treatment, highlights the practical utility of point-of-care aMMP-8 test technologies for the real-time and online diagnosis of periodontitis, as well as for monitoring the periodontal treatment outcome [18,23,25,29]. The FDA/USA and EU-approved aMMP 8 POC test is commercially and globally available to any dental practitioner or oral hygienist [23,25,32]. We hereby present the visual aMMP-8 test, the aMMP-8 POCT quantitated by ORALyzer, the aMMP-8 RFU activity assay, the total MMP-8 assay, and other potentially related biomarkers (MPO, PMN elastase, tissue inhibitor of MMPs [TIMP]-1, calprotectin, and interleukin-6) and clinical data of 13 adult patients with severe (stage III/IV, grade B/C) chronic periodontitis to diagnose and monitor the anti-infective scaling and root planing of adult patients with chronic periodontitis of stage III/IV-grade b/c, with the sample size (n = 13) representing globally any dentist’s or oral hygienist’s set of patients in one day. The present study therefore aimed to compare potential mouthrinse periodontitis biomarkers (the aMMP-8 POCT, the aMMP-8 RFU activity assay, and total MMP-8) and related biomarkers (MPO, PMN elastase, calprotectin, and interleukin-6) in periodontal disease diagnosis and in monitoring the effects of nonsurgical anti-infective periodontal therapy. Additionally, we compared the cut-off values (10, 20, and 25 ng/mL) previously used for the aMMP-8 POCT technology [18,21,24,25,26,27,28,29].

## 2. Results

**Figure 1:**
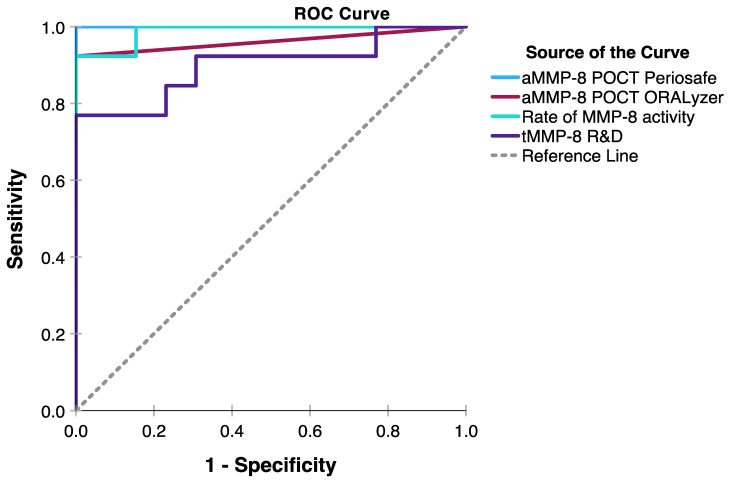
The diagnostic performance of four different MMP-8 analysis methods targeting either active or total MMP-8 species illustrated by ROC analysis in 13 patients with stage III/IV-grade B/C periodontitis and 13 periodontally and systemically healthy controls.

Figure 1 and Table 1 present the diagnostic performance of the aMMP-8 POCT, the rate of aMMP-8 RFU activity, tMMP-8, MPO, PMN elastase, TIMP-1, calprotectin, and IL-6 to discriminate patients with periodontitis and healthy controls. The best performance was shown by the aMMP-8 POCT, and next were the rate of MMP-8 RFU activity, MPO, PMN elastase, and tMMP-8, while TIMP-1, calprotectin, and IL-6 had much lower performance. Furthermore, it was clearly noted that cut-off value of 20 ng/mL for the aMMP-8 POCT was preferable to 25 ng/mL and 10 ng/mL.

### Periodontal Anti-Infective Treatment Effect

**Figure 2:**
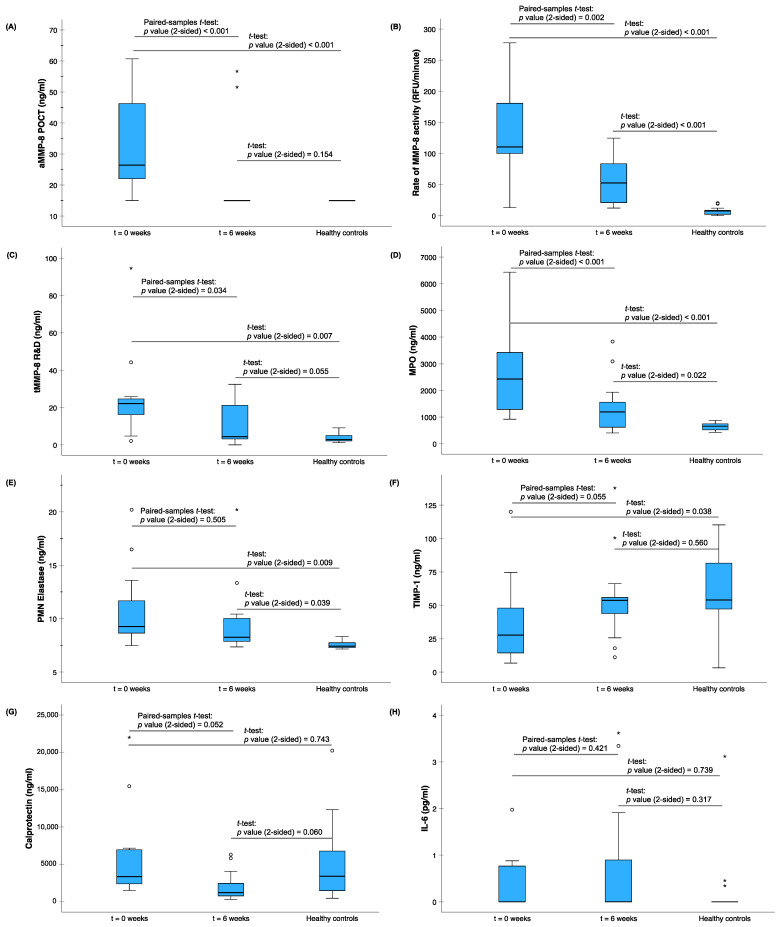
The effects of anti-infective treatment in 13 patients with periodontitis on (**A**) the aMMP-8 POCT (ng/mL) (ORALyzer), (**B**) the MMP-8 activity assay (RFU per minute), (**C**) total MMP-8 (ng/mL) R&D systems ELISA, (**D**) MPO (ng/mL), (**E**) PMN elastase (ng/mL), (**F**) TIMP-1 (ng/mL), (**G**) calprotectin (ng/mL), and (**H**) IL-6 (pg/mL) vs. levels in 13 healthy controls. The patients were examined at baseline visit t0 and at 1st recall visit t1 (6 weeks). The differences in the clinical parameters between t0 and t1 were tested with a paired-samples *t*-test (2-sided), (**A**) *p* < 0.001, (**B**) *p* = 0.002, (**C**) *p* = 0.034, (**D**) *p* < 0.001, (**E**) *p* = 0.505, (**F**) *p* = 0.055, (**G**) *p* = 0.052, and (**H**) *p* = 0.421, and between healthy controls and t0 and t1 (*t*-test, Bonferroni corrected), (**A**) *p* < 0.001 and *p* = 0.154, (**B**) *p* < 0.001 and *p* < 0.001, (**C**) *p* = 0.007 and *p* = 0.055, (**D**) *p* < 0.001 and *p* = 0.022, (**E**) *p* = 0.009 and *p* = 0.039, (**F**) *p* = 0.038 and *p* = 0.560, (**G**) *p* = 0.743 and *p* = 0.060, and (**H**) *p* = 0.739 and *p* = 0.317, respectively. Asterisk (*) and circle (o) represent outliers of more than 3 times the interquartile range and between 1.5 and 3 times the interquartile range, respectively.

**Figure 3:**
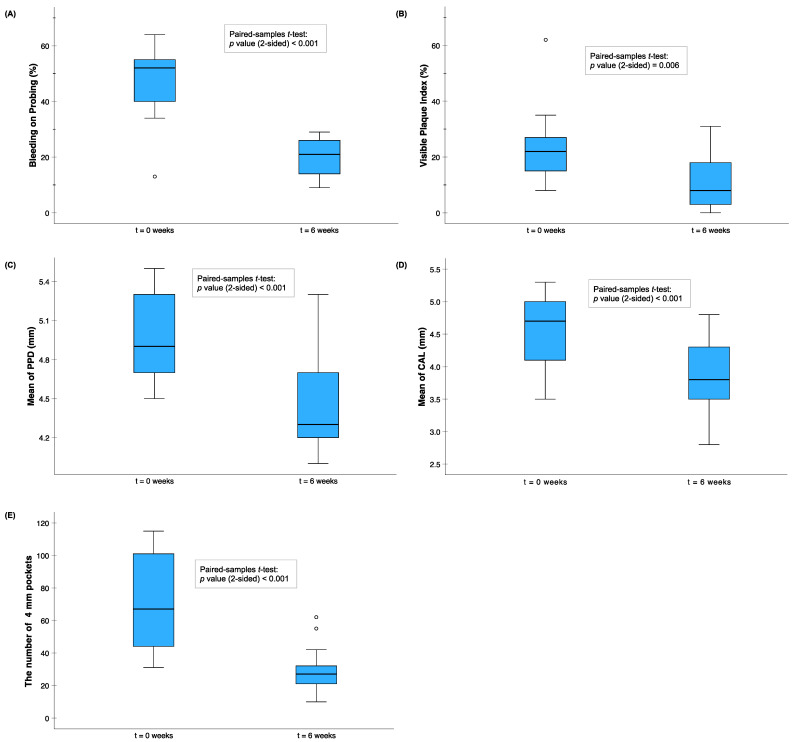
The treatment effects of the periodontal anti-infective treatment on the recorded clinical periodontal parameters in 13 patients with periodontitis: (**A**) bleeding on probing (%), (**B**) visible plaque index (%), (**C**) mean of PPD (mm), (**D**) mean of CAL (mm), and (**E**) the number of at least 4 mm periodontal pockets. The patients were examined at base level t0 and at 1st recall visit t1 (6 weeks). The differences in the clinical parameters between t0 and t1 were tested with a paired-samples *t*-test (2-sided): (**A**) *p* < 0.001, (**B**) *p* = 0.006, (**C**) *p* < 0.001, (**D**) *p* < 0.001, and (**E**) *p* < 0.001. Circle (o) represents outliers of between 1.5 and 3 times the interquartile range.

The effects of anti-infective treatment (scaling and root planning) in 13 periodontitis patients were monitored by aMMP-8, the rate of aMMP-8 RFU activity, tMMP-8, MPO, PMN elastase, TIMP-1, calprotectin, and IL-6 as well as by clinical periodontal parameters (Figure 2 and Figure 3). The significant decrease in the levels of aMMP-8, the rate of aMMP-8 RFU activity, tMMP-8, and MPO, as well as in the levels of bleeding on probing, the visible plaque index, the mean of PPD, the mean of CAL, and the number of at least 4 mm periodontal pockets, showed a positive and beneficial treatment effect in these patients with periodontitis. There was also a decrease in the levels of PMN elastase and calprotectin and an increase in the levels of TIMP-1, but they did not reach the level of significance. Finally, there was a significant difference in the levels of aMMP-8, the rate of MMP-8 activity, tMMP-8, MPO, PMN elastase, and TIMP-1 between the base level of (untreated) patients with periodontitis and 13 periodontally and systemically healthy controls.

## 3. Discussion

We demonstrated here the straight-forward and practical usefulness of the mouthrinse aMMP-8 POCT technology for online and real-time chair-side diagnosis and treatment for monitoring the number of patients likely to be seen by a dentist, periodontist, or oral hygienist (n = 13) with severe, i.e., stage III/IV-grade b/c, periodontitis in one day. Every dentist, periodontist, or oral hygienist can personally and conveniently calibrate themselves this way. Because aMMP-8 predictively makes invisible disease activity visible and thus predictively alarms the worsening of periodontal health status and behavior of patients, it provides in good time early messages about these developing pathological events to the dentist and/or oral hygienist [23,25,29,31,32]. The aMMP-8 POCT thus can additionally be utilized to motivate the patients as well as the dentists and oral hygienists [18,23,31]. Successful anti-infective treatment can be conveniently monitored online and in real time by the visual and/or by the reader-quantitated aMMP-8 POCT. The results in this study are in agreement and further extend previous studies in that regard and exhibit the eventual benefits of utilizing the aMMP-8 POCT analysis for the discrimination and monitoring of periodontal health and disease [23,25,31,32,33,34,35,36]. Furthermore, our present findings are in accordance with and further extend earlier studies linking elevated oral aMMP-8 but not total MMP-8 to active and progressive stages of periodontal and peri-implant diseases [18,29,34,37,38,39,40,41,42]. The aMMP-8 POCT, MPO, PMN elastase, and the aMMP-8 RFU catalytic activity assay were superior relative to the other tested potential and related biomarkers, including total MMP-8, calprotectin, TIMP-1, and interleukin-6. Furthermore, the aMMP-8 POCT and the independent aMMP-8 RFU catalytic activity assay correlated well with each other in this study and in accordance with further extending previous studies [18,37]. aMMP-8, MPO, and PMN elastase eventually form a pro-oxidative and proteolytic tissue cascade in stage III/IV-grade b/c periodontitis that can conveniently be identified by the aMMP-8 POCT in 5 min. Noteworthy, anti-oxidative, reactive oxygen species scavengers and protease inhibitors such as tetracycline/doxycycline, chlorhexidine, and phenolic compounds of fermented lingonberry juice eventually can be utilized as therapeutic targets. Also, MPO, a pro-oxidative activator of MMP-8, was recorded in this study to be an efficient biomarker of periodontitis. In that regard, the present study showed that MPO and PMN elastase persisted in elevated levels in patients with periodontitis compared to healthy controls after the treatment phase, which may suggest the eventual elevation of aMMP-8 levels and the eventual elevation of periodontitis disease activity in the future that can be monitored by the aMMP-8 POCT in the periodontitis treatment and maintenance phases.

We recommend the use of the validated, simple mouthrinse/oral fluid aMMP-8 POCT assay [18,23,24,25,26,27,28,29,31] to be used by every dentist or oral hygienist in their daily/weekly diagnosing and monitoring of periodontitis and its treatment and maintenance. As a very sensitive biomarker, it is additionally suitable for the monitoring of periodontal disease in patients with systemic diseases, such as subclinical diabetes, metabolic syndrome, obesity, atherosclerosis, cancers, ophthalmological diseases, and Alzheimer’s disease, as well as in patients with head and neck cancer who can rapidly develop oral-mucositis-related and periodontitis side effects [29,35,43,44,45]. Furthermore, we suggest the inclusion of aMMP-8 as an indicator of staging and grading of the clinical attachment loss/bone loss related to active periodontal degeneration, i.e., collagen destruction in these systemic pathologies. In this regard, the utilization of a promising aMMP-8 POCT needs further additional interdisciplinary and independent validations.

Our results, based on an ROC curve analysis of the tested biomarkers, i.e., the visual aMMP-8 POCT, the aMMP-8 POCT quantified by ORALyzer, MPO, the aMMP-8 RFU activity assay, and tMMP-8-, PMN-elastase-, TIMP-1-, calprotectin-, and interleukin-6-ELISAs, revealed that regarding both online, real-time chair-side point-of-care (i) diagnostics and (ii) the monitoring of anti-infective scaling and root planing treatment, that both the visual ([+] > 20 ng/mL, [−] ≤ 20 ng/mL) aMMP-8 and the aMMP-8 ORALyzer-quantitated POCT together with the MMP-8 oxidative activator MPO were the most precise biomarkers for periodontitis. The second most efficient analysis was the independent aMMP-8 RFU catalytic activity assay [18,38]. The total MMP-8 ELISA, in accordance with previous studies [44,45,46,47,48,49], was not as good as the above two independent and specific aMMP-8 assays and MPO. The PMN-elastase- and calprotectin-ELISAs were less efficient, and TIMP-1 and interleukin-6 were incapable to be utilized as liable biomarkers for the diagnostic discrimination and monitoring of the anti-infective scaling and root planing treatment.

Regarding the cut-off values for aMMP-8, three different cut-offs (10, 20, and 25 ng/mL) were presented and used for the aMMP-8 POCT [18,21,24,25,26,27,28,29,35,36]. We demonstrated here that the 20 ng/mL cut-off [18,25,29,35] was the most optimal for successful chair-side diagnosis and for monitoring the beneficial clinical effects of the anti-infective scaling and root planing treatment [50]. We have previously shown [36] that a cut-off of 10 ng/mL [24,26,27,28] is not suitable for the online, real-time monitoring of the beneficial effects of the anti-infective scaling and root planning treatment of periodontitis.

Our earlier studies [33,34,35,36] have repeatedly and consistently revealed that there exists a large effect size regarding aMMP-8, strongly suggesting that there is not always a need for the large number (n) of patients and healthy controls to obtain statistically significant results. The apparent inability as also recorded in this study (Table 1) of the aMMP-8 POCT with a cut-off of 20 ng/mL to result in false positives [18,29,35,36] eventually contributes to this beneficial property and outcome of the aMMP-8 POCT performance recorded with a cut-off of 20 ng/mL [18,29,35,51,52]. With this background, our relatively small number of patients (n = 13) and healthy controls (n = 13) should in fact be regarded as sufficient, and accordingly therefore should not be regarded as a limitation. Noteworthy, collagenolytic and catalytically competent aMMP-8, but not non-collagenolytic and catalytically non-competent total latent pro-MMP-8, is a precise biomarker to detect, monitor, reflect, and indicate progressive collagenolytic periodontitis in its clinically active tissue-destructive phases [12,23,25,32,38,39,40,51,53]. Yet, utilizing healthy dental students may be a limitation in this study, as the patients with periodontitis are much older and with different smoking histories (smokers, non-smokers, ex-smokers, etc.) than the control patients, the dental students. Thus, the biomarker levels may potentially be partly affected by the age- and lifestyle-related differences independent from periodontitis. This should be addressed in future studies comparing biomarker levels in different types of periodontally and systemically healthy patients.

Previous studies have revealed that aMMP-8 is not synonymous to total MMP-8 in periodontitis and peri-implantitis oral fluid diagnostics [25]. In addition, total MMP-8 can fail or at least is clearly less efficient in periodontitis and peri-implantitis oral fluid diagnostics biomarker and treatment monitoring [45,46,47,48]. Our present findings further support and extend this conjuncture [45,46,47,48]. aMMP-8, measured by various independent and specific immunological and catalytic activity assays as the oral fluid diagnostic biomarker and/or indicator of active periodontitis phases or in treatment monitoring, has never failed [35,36,37,38,39,47,51]. Overall, the aMMP-8 POCT with a cut-off of 20 ng/mL [18,25,29] as also revealed by this study is a very suitable biomarker for chair-side diagnostics and treatment monitoring of chronic adult periodontitis. Every periodontist, dentist, and oral hygienist can calibrate themselves and their number of patients in one day by the personalized medicine-manner-utilizing aMMP-8 POCT as described in this paper. This study further revealed that the aMMP-8 POCT can be conveniently utilized by dentists and oral hygienists to motivate their patients by the current state of periodontitis disease activity to better adhere to the treatment and maintenance phase. Additional interdisciplinary studies with the aMMP-8 POCT are recommended.

## 4. Materials and Methods

### 4.1. Study Population, Sample Size, and Design

Previous studies have suggested good predictive power for discriminating periodontitis and periodontal health using the aMMP-8 POCT in gingival crevicular fluid (GCF) and peri-implant sulcular fluid (PISF) [31,32,33,34]. One ROC curve power calculation was performed (R version 3.6.3 (29 February 2020) and pROC package 1.16.2), which revealed that a total of 13 patients with periodontitis and 13 healthy controls were needed to reach 80% power with an AUC of 0.80, a significance level of 5%, and kappa of 1.

A total of 13 Finnish adult patients visiting the Helsinki University Hospital Oral and Maxillofacial Diseases Clinic for their stage III/IV-grade b/c periodontal problems were recruited in the present study as previously described [18,30,37]. The study was approved by the Helsinki University and Stockholm Ethics Committees (106§/26.06.2019; dnro HUS/1271/2019; 2016-08-24/2016/1:8 and 2016-1-24; Dnr 2016/1410-31/1). This study was conducted according to the principles of the Declaration of Helsinki. All recruited participants provided oral and written consent. The inclusion criteria for this study were interdental clinical attachment loss of at least 5 mm (at the site of greatest loss), radiographic bone loss that extended to the mid-third of a root and beyond, and tooth loss because of periodontitis that was ≤4 teeth (stage III periodontitis) and ≥5 teeth (stage IV periodontitis). aMMP-8 was consistently elevated (>20 ng/mL, visual [+, ++, +++]) in all adult patients with chronic periodontitis, indicating grade b/c [18,36]. The patients had not received any antimicrobial or MMP-8 inhibitory low-dose doxycycline, regular-dose doxycycline, bisphosphonates, or chlorhexidine medications [6,18]. Patients with acquired immune deficiency syndrome (AIDS), uncontrolled diabetes (HbA1c > 7), and other immune-system-related chronic diseases (Crohn’s disease, etc.) as well as oral neoplasms and patients under chemotherapy treatment were excluded from this study. Pregnant or lactating females and individuals who had received periodontal treatment or systemic antibiotics within the last year were also excluded. A total of 13 systemically and periodontally healthy Finnish dental students from the University of Helsinki, Finland, participated as healthy controls (HCs) [18,36]; all HC dental students’ aMMP-8 POCT results were ≤20 ng/mL, thus they were visually [−] [18,29,36].

The patient characteristics are presented in Table 2.

### 4.2. Periodontal Examination and Anti-Infective Treatment Procedures

Comprehensive full-mouth clinical recordings and periodontal examinations were performed at baseline (t0) and 6 weeks (t1) following periodontal treatment (anti-infective scaling and root planing) by a single periodontist (N.R.). In the examination, a WHO Probe 550B periodontal probe was used for measuring probing depths (PDs) at six sites of each tooth, which was followed by determining the BOP percentage. Furthermore, the visible plaque index (VPI) was recorded for each patient by assigning a score of 0–3 to each surface and was used for calculating the average oral plaque score. Clinical attachment levels (CALs) were determined as described [18,36]. The anti-infective full-mouth scaling and root planing treatment procedures were performed after clinical full-mouth recordings and the aMMP-8 POCT, along with oral hygiene instructions for 13 patients with stage III/IV-grade b/c periodontitis at baseline (t0) and 6 weeks (t1) [18]. The 13 systemically and periodontally healthy dental students (23–25 years old), who were enrolled as healthy controls (HC), had aMMP-8 POCT and related similar mouthrinse biomarker sample collections and similar full-mouth clinical examinations [18,36].

### 4.3. Chair-Side Quantitative POC aMMP-8 Analyses

aMMP-8 levels were measured online by rapid POC chair-side aMMP-8 kits (Periosafe^®^, Dentognostics GmbH, Solingen, Germany) and were visually ([+] > 20 ng/mL, [−] ≤ 20 ng/mL) and real-time, online quantitated by ORALyzer^®^ (Dentognostics GmbH, Solingen, Germany) in the periodontitis patient group (n = 13) and the healthy control (HC) group of 13 systemically and periodontally healthy dental students according to the instructions of the manufacturer [18,29,36]. We used the aMMP-8 POCT industrially fabricated with a cut-off of 20 ng/mL [18,29,34,35]. Other cut-off values (10 and 25 ng/mL) used in previous publications [24,25,27,29] were also assessed. The cut-off values for the aMMP-8 RFU activity assay, MPO, PMN elastase, total MMP-8, TIMP-1, calprotectin, and interleukin-6 were adjusted by the computer to levels representing the highest value detected in HCs. Any remaining oral mouthrinse fluid was transferred to Eppendorf tubes and stored at −70 °C for further laboratory analysis of additional potential biomarkers (the aMMP-8 RFU activity assay, tMMP-8, TIMP-1, MPO, PMN elastase, calprotectin, interleukin-6) by the RFU activity assay and ELISA assays as described previously [18,37].

### 4.4. aMMP-8 Activity Assay Using Relative Fluorescence Units/min (RFU)

An MMP-8 activity assay for measuring MMP-8 activity was adapted from the protocol of Mc Crudden et al. (2017) with slight modifications, as described in detail previously in McCrudden et al. and Aji et al. [18,37]. In short, an aMMP-8-specific antibody (Merck Millipore, Watford, UK) was used to coat the wells of Greiner^®^ 96-well black high-binding plates (Merck, Darmstadt, Germany) to capture aMMP-8 selectively. The activity of the captured aMMP-8 was measured using a fluorogenic substrate on a microtiter plate reader (Genios, Tecan, Reading, UK) using Magellan software Version 7.2 (Tecan, Reading, UK). The results were expressed in relative fluorescence units per minute (RFU/min) [18,37].

### 4.5. Statistical Analyses

A receiver operating characteristic (ROC) analysis was performed to evaluate the diagnostic accuracy of potential periodontitis biomarkers (the aMMP-8 POCT, the aMMP-8 RFU activity assay, total MMP-8, MPO, PMN elastase, calprotectin, and interleukin-6). The optimal cut-off points for the levels of biomarkers were determined by Youden’s Index. The treatment effect of periodontal anti-infective treatment to the levels of the biomarkers and to the recorded clinical periodontal parameters (BOP, visible plaque index, mean of PPD, mean of CAL, and the number of at least 4 mm periodontal pockets) were assessed by a paired-samples *t*-test. Finally, the biomarker levels of the base level of (untreated) patients with periodontitis and 13 periodontally and systemically healthy controls were compared by *t*-test (Bonferroni corrected). Statistical significance was considered if *p* < 0.05 (2-sided).

## 5. Conclusions

aMMP-8, MPO, and PMN elastase eventually form a proteolytic and pro-oxidative tissue destruction cascade in periodontitis, potentially representing a therapeutic target. The active MMP-8 POCT identifies this periodontitis tissue destruction cascade in 5 min. The aMMP-8 POCT is the most efficient and precise discriminator between periodontal health and disease. The aMMP-8 POC test can be effectively used by dental professionals in their dental practices for the online and real-time diagnosis and monitoring of periodontal disease and to educate their patients and encourage good oral practices.

## Figures and Tables

**Table 1:** The diagnostic performance of several biomarker candidates for periodontitis using the optimal cut-off values calculated by Youden’s index in 13 patients with periodontitis and 13 healthy controls. ORALyzer was evaluated with cut-off values of 20 ng/mL (by Youden’s index) and 25 ng/mL.

Biomarker	ROC AUC (95% Confidence Interval)	Cut-Off Points (By Youden’s Index)		Periodontitis	Healthy	OR	Sensitivity	Specificity	FN	FP	ACC	MCC
aMMP-8 PerioSafe	1.000 (1.000–1.000)	Positive test	Test positive	13	0	729	100.0%	100.0%	0.0%	0.0%	100.0%	1.000
			Test negative	0	13							
aMMP-8 ORALyzer	0.962 (0.875–1.000)	20 ng/mL	≥20 ng/mL	12	0	225	92.3%	100.0%	7.1%	0.0%	96.2%	0.926
			<20 ng/mL	1	13							
	–	–	≥25 ng/mL	8	0	41.7	61.5%	100.0%	27.8%	0.0%	80.8%	0.667
			<25 ng/mL	5	13							
Rate of MMP-8 activity	0.988 (0.958–1.000)	37.21 RFU/min	≥37.21 RFU/min	12	0	225	92.3%	100.0%	7.1%	0.0%	96.2%	0.926
			<37.21 RFU/min	1	13							
tMMP-8 R&D	0.899 (0.770–1.000)	12.70 ng/mL	≥12.70 ng/mL	10	0	81	76.9%	100.0%	18.8%	0.0%	88.5%	0.791
			<12.70 ng/mL	3	13							
MPO	1.000 (1.000–1.000)	896.91 ng/mL	≥896.91 ng/mL	13	0	729	100.0%	100.0%	0.0%	0.0%	100.0%	1.000
			<896.91 ng/mL	0	13							
PMN Elastase	0.959 (0.888–1.000)	8.38 ng/mL	≥8.38 ng/mL	11	0	124.2	84.6%	100.0%	13.3%	0.0%	92.3%	0.856
			<8.38 ng/mL	2	13							
TIMP-1	0.231 (0.031–0.431)	42.04 ng/mL	<42.04 ng/mL	9	2	9.7	69.2%	84.6%	26.7%	18.2%	76.9%	0.545
			≥42.04 ng/mL	4	11							
Calprotectin	0.574 (0.347–0.801)	1494.5 ng/mL	≥1494.5 ng/mL	13	9	12.8	100.0%	30.8%	0.0%	40.9%	65.4%	0.426
			<1494.5 ng/mL	0	4							
IL-6	0.621 (0.400–0.842)	0.50 pg/mL	≥0.50 pg/mL	5	1	5.4	38.5%	92.3%	40.0%	16.7%	65.4%	0.365
			<0.50 pg/mL	8	12							

OR: odds ratio; FN: false negatives; FP: false positives; ACC: accuracy; MCC: Matthew’s correlation coefficient.

**Table 2:** Patient characteristics.

Patient Characteristics		Patients with Periodontitis N = 13	Healthy Dental Student Controls N = 13
Age (in years)	Mean ± standard deviation	52 ± 10	25 ± 4
Gender n (%)	Male	10 (77%)	3 (23%)
	Female	3 (23%)	10 (77%)
Systemic status n (%)	Healthy	7 (54%)	13 (100%)
	Cardiovascular diseases	1 (8%)	0 (0%)
	Hypertension	5 (38%)	0 (0%)
	Hypercholesterolemia	2 (16%)	0 (0%)
	Type I diabetes	1 (8%)	0 (0%)
	Asthma	1 (8%)	0 (0%)
Smoking n (%)	Yes *	3 (23%)	0 (0%)
	No	6 (46%)	13 (100%)
	Ex-smoker ˆ	4 (31%)	0 (0%)

*: All smoked 20–40 cigarettes per day. ˆ: All had stopped more than 2 years ago.

## Data Availability

Data supporting reported that the results can be obtained from the authors on request.

## References

[1] Kinane D.F., Stathopoulou P.G., Papapanou P.N. (2017). Periodontal diseases. Nat. Rev. Dis. Primers.

[2] Papapanou P.N., Sanz M., Buduneli N., Dietrich T., Feres M., Fine D.H., Flemmig T.F., Garcia R., Giannobile W.V., Graziani F. (2018). Periodontitis: Consensus report of workgroup 2 of the 2017 World Workshop on the Classification of Periodontal and Peri-Implant Diseases and Conditions. J. Periodontol..

[3] Tonetti M.S., Greenwell H., Kornman K.S. (2018). Staging and grading of periodontitis: Framework and proposal of a new classification and case definition. J. Periodontol..

[4] Sanz M., Herrera D., Kebschull M., Chapple I., Jepsen S., Berglundh T., Sculean A., Tonetti M.S., On behalf of the EFP Workshop Participants and Methodological Consultants (2020). Treatment of stage I-III periodontitis—The EFP S3 level clinical practice guideline. J. Clin. Periodontol..

[5] Herrera D., Sanz M., Kebschull M., Jepsen S., Sculean A., Berglundh T., Papapanou P.N., Chapple I., Tonetti M.S., On behalf of the EFP Workshop Participants and Methodological Consultant (2022). Treatment of stage IV periodontitis: The EFP S3 level clinical practice guideline. J. Clin. Periodontol..

[6] Tschesche H., Wenzel H., Rawlings N.D., Salvesen G. (2013). Chapter 153—Neutrophil Collagenase. Handbook of Proteolytic Enzymes.

[7] Weiss S.J. (1989). Tissue destruction by neutrophils. N. Engl. J. Med..

[8] Dieffenbach P.B., Mallarino Haeger C., Rehman R., Corcoran A.M., Coronata A.M.F., Vellarikkal S.K., Chrobak I., Waxman A.B., Vitali S.H., Sholl L.M. (2021). A Novel Protective Role for Matrix Metalloproteinase-8 in the Pulmonary Vasculature. Am. J. Respir. Crit. Care Med..

[9] Khatwa U.A., Kleibrink B.E., Shapiro S.D., Subramaniam M. (2010). MMP-8 promotes polymorphonuclear cell migration through collagen barriers in obliterative bronchiolitis. J. Leukoc. Biol..

[10] Boelen G.J., Boute L., d’Hoop J., EzEldeen M., Lambrichts I., Opdenakker G. (2019). Matrix metalloproteinases and inhibitors in dentistry. Clin. Oral Investig..

[11] De Morais E.F., Pinheiro J.C., Leite R.B., Santos P.P.A., Barboza C.A.G., Freitas R.A. (2018). Matrix metalloproteinase-8 levels in periodontal disease patients: A systematic review. J. Periodontal Res..

[12] Luchian I., Goriuc A., Sandu D., Covasa M. (2022). The Role of Matrix Metalloproteinases (MMP-8, MMP-9, MMP-13) in Periodontal and Peri-Implant Pathological Processes. Int. J. Mol. Sci..

[13] Arias-Bujanda N., Regueira-Iglesias A., Balsa-Castro C., Nibali L., Donos N., Tomás I. (2019). Accuracy of single molecular biomarkers in gingivael crevicular fluid for the diagnosis of periodontitis: A systematic review and metaanalysis. J. Clin. Periodontol..

[14] Gupta N., Gupta N.D., Gupta A., Khan S., Bansal N. (2015). Role of salivary matrix metalloproteinase-8 (MMP-8) in chronic periodontitis diagnosis. Front. Med..

[15] Chou Y.-H., Ho Y.-P., Lin Y.-C., Hu K.-F., Yang Y.-H., Ho K.-Y., Wu Y.-M., Hsi E., Tsai C.-C. (2011). MMP-8-799 C>T genetic polymorphism is associated with the susceptibility to chronic and aggressive periodontitis in Taiwanese. J. Clin. Periodontol..

[16] Gul S.S., Abdulkareem A.A., Sha A.M., Rawlinson A. (2020). Diagnostic Accuracy of Oral Fluids Biomarker Profile to Determine the Current and Future Status of Periodontal and Peri-Implant Diseases. Diagnostics.

[17] Yuan C., Liu X., Zheng S. (2018). Matrix metalloproteinase-8 levels in oral samples as a biomarker for periodontitis in the Chinese population: An observational study. BMC Oral Health.

[18] Aji N.R.A.S., Yucel-Lindberg T., Räisänen I.T., Kuula H., Nieminen M.T., Mc Crudden M.T.C., Listyarifah D., Lundmark A., Lundy F.T., Gupta S. (2024). In Vivo Regulation of Active Matrix Metalloproteinase-8 (aMMP-8) in Periodontitis: From Transcriptomics to Real-Time Online Diagnostics and Treatment Monitoring. Diagnostics.

[19] Ramenzoni L.L., Hofer D., Solderer A., Wiedemeier D., Attin T., Schmidlin P.R. (2021). Origin of MMP-8 and Lactoferrin levels from gingival crevicular fluid, salivary glands and whole saliva. BMC Oral Health.

[20] Choi D.H., Moon I.S., Choi B.K., Paik J.W., Kim Y.S., Choi S.H., Kim C.K. (2004). Effects of sub-antimicrobial dose doxycycline therapy on crevicular fluid MMP-8, and gingival tissue MMP-9, TIMP-1 and IL-6 levels in chronic periodontitis. J. Periodontal Res..

[21] Izadi Borujeni S., Mayer M., Eickholz P. (2015). Activated matrix metalloproteinase-8 in saliva as diagnostic test for periodontal disease? A case–control study. Med. Microbiol. Immunol..

[22] Johnson N., Ebersole J.L., Kryscio R.J., Danaher R.J., Dawson D., Al-Sabbagh M., Miller C.S. (2016). Rapid assessment of salivary MMP-8 and periodontal disease using lateral flow immunoassay. Oral Dis..

[23] Sahni V. (2024). Point of care technology for screening and referrals. Br. Dent. J..

[24] Deng K., Pelekos G., Jin L., Tonetti M.S. (2021). Diagnostic accuracy of a point-of-care aMMP-8 test in the discrimination of periodontal health and disease. J. Clin. Periodontol..

[25] Sahni V., Räisänen I.T., Sorsa T. (2024). MMP-8 and active MMP-8 (aMMP-8) are not synonymous in periodontal disease diagnostics. J. Clin. Periodontol..

[26] Deng K., Wei S., Xu M., Shi J., Lai H., Tonetti M.S. (2022). Diagnostic accuracy of active matrix metalloproteinase-8 point-of-care test for the discrimination of periodontal health status: Comparison of saliva and oral rinse samples. J. Periodontal Res..

[27] Deng K., Zonta F., Yang H., Pelekos G., Tonetti M.S. (2023). Development of a machine learning multiclass screening tool for periodontal health status based on non-clinical parameters and salivary biomarkers. J. Clin. Periodontol..

[28] Wei S., Lin T., Sáenz-Ravello G., Gao H., Zhang Y., Tonetti M.S., Deng K. (2024). Diagnostic accuracy of salivary active matrix metalloproteinase (aMMP)-8 point-of-care test for detecting periodontitis in adults: A systematic review and meta-analysis. J. Clin. Periodontol..

[29] Umeizudike K.A., Aji N.R.A.S., Niskanen K., Rantala I., Sakellari D., Grigoriadus A., Patila T., Gupta S., Sorsa T., Raisanen I.T. (2024). Prediabetes associates with matrix metalloproteinase-8 activation and contributes to the rapid destruction of periodontal tissue. Eur. J. Dent..

[30] Nędzi-Góra M., Kostrzewa-Janicka J., Górska R. (2014). Elastase and metalloproteinase-9 concentrations in saliva in patients with chronic periodontitis. Cent. Eur. J. Immunol..

[31] Gellibolian R., Miller C.S., Markaryan A.N., Weltman R.L., van Dyke T.E., Ebersole J.L. (2022). Precision periodontics: Quantitative measures of disease progression. J. Am. Dent. Assoc..

[32] Zalewska E.A., Ławicka R., Grygorczuk P., Nowosielska M., Kicman A., Ławicki S. (2024). Importance of Metalloproteinase 8 (MMP-8) in the Diagnosis of Periodontitis. Int. J. Mol. Sci..

[33] Hernández M., Baeza M., Räisänen I.T., Contreras J., Tervahartiala T., Chaparro A., Sorsa T., Hernández-Ríos P. (2021). Active MMP-8 Quantitative Test as an Adjunctive Tool for Early Diagnosis of Periodontitis. Diagnostics.

[34] Xanthopoulou V., Räisänen I.T., Sorsa T., Tortopidis D., Sakellari D. (2024). Diagnostic value of aMMP-8 and azurocidin in peri-implant sulcular fluid as biomarkers of peri-implant health or disease. Clin. Exp. Dent. Res..

[35] Thomas J.T., Joseph B., Varghese S., Thomas N.G., Kamalasanan Vijayakumary B., Sorsa T., Anil S., Waltimo T. (2024). Association between metabolic syndrome and salivary MMP-8, myeloperoxidase in periodontitis. Oral Dis..

[36] Keskin M., Rintamarttunen J., Gülçiçek E., Räisänen I.T., Gupta S., Tervahartiala T., Pätilä T., Sorsa T. (2023). A Comparative Analysis of Treatment-Related Changes in the Diagnostic Biomarker Active Metalloproteinase-8 Levels in Patients with Periodontitis. Diagnostics.

[37] Mc Crudden M.T.C., Irwin C.R., El Karim I., Linden G.J., Lundy F.T. (2017). Matrix metalloproteinase-8 activity in gingival crevicular fluid: Development of a novel assay. J. Periodontal Res..

[38] Mancini S., Romanelli R., Laschinger C.A., Overall C.M., Sodek J., McCulloch C.A. (1999). Assessment of a novel screening test for neutrophil collagenase activity in the diagnosis of periodontal diseases. J. Periodontol..

[39] Romanelli R., Mancini S., Laschinger C., Overall C.M., Sodek J., McCulloch C.A. (1999). Activation of neutrophil collagenase in periodontitis. Infect. Immun..

[40] Lee W., Aitken S., Sodek J., McCulloch C.A.G. (1995). Evidence of a direct relationship between neutrophil collagenase activity and periodontal tissue destruction in vivo: Role of active enzyme in human periodontitis. J. Periodontal Res..

[41] Gangbar S., Overall C.M., McCulloch C.A.G., Sodek J. (1990). Identification of polymorphonuclear leukocyte collagenase and gelatinase activities in mouthrinse samples: Correlation with periodontal disease activity in adult and juvenile periodontitis. J. Periodontal Res..

[42] Guarnieri R., Reda R., Zanza A., Miccoli G., Nardo D.D., Testarelli L. (2022). Can Peri-Implant Marginal Bone Loss Progression and a-MMP-8 Be Considered Indicators of the Subsequent Onset of Peri-Implantitis? A 5-Year Study. Diagnostics.

[43] Sadrameli M., Bathini P., Alberi L. (2020). Linking mechanisms of periodontitis to Alzheimer’s disease. Curr. Opin. Neurol..

[44] Sanz M., Del Castillo A.M., Jepsen S., Gonzalez-Juanatey J.R., D’Aiuto F., Bouchard P., Chapple I., Dietrich T., Gotsman I., Graziani F. (2020). Periodontitis and Cardiovascular Diseases: Consensus Report. Glob. Heart.

[45] Gasparoni L.M., Alves F.A., Holzhausen M., Pannuti C.M., Serpa M.S. (2021). Periodontitis as a risk factor for head and neck cancer. Med. Oral Patol. Oral Cir. Bucal.

[46] Verhulst M.J.L., Teeuw W.J., Bizzarro S., Muris J., Su N., Nicu E.A., Nazmi K., Bikker F.J., Loos B.G. (2019). A rapid, non-invasive tool for periodontitis screening in a medical care setting. BMC Oral Health.

[47] Romero-Castro N.S., Vázquez-Villamar M., Muñoz-Valle J.F., Reyes-Fernández S., Serna-Radilla V.O., García-Arellano S., Castro-Alarcón N. (2020). Relationship between TNF-α, MMP-8, and MMP-9 levels in gingival crevicular fluid and the subgingival microbiota in periodontal disease. Odontology.

[48] Wang H.L., Garaicoa-Pazmino C., Collins A., Ong H.S., Chudri R., Giannobile W.V. (2016). Protein biomarkers and microbial profiles in peri-implantitis. Clin. Oral Implant Res..

[49] Wohlfahrt J.C., Aass A.M., Granfeldt F., Lyngstadaas S.P., Reseland J.E. (2014). Sulcus fluid bone marker levels and the outcome of surgical treatment of peri-implantitis. J. Clin. Periodontol..

[50] Morrison E.C., Ramfjord S.P., Hill R.W. (1980). Short-term effects of initial, nonsurgical periodontal treatment (hygienic phase). J. Clin. Periodontol..

[51] AlMoharib H.S., AlRowis R., AlMubarak A., Waleed Almadhoon H., Ashri N. (2023). The relationship between matrix metalloproteinases-8 and peri-implantitis: A systematic review and meta-analysis. Saudi Dent. J..

[52] Atanasova T., Stankova T., Bivolarska A., Vlaykova T. (2023). Matrix Metalloproteinases in Oral Health-Special Attention on MMP-8. Biomedicines.

[53] Overall C.M., Sodek J., McCulloch C.A., Birek P. (1991). Evidence for polymorphonuclear leukocyte collagenase and 92-kilodalton gelatinase in gingival crevicular fluid. Infect Immun..

